# A physiotherapy group exercise and self-management approach to improve physical activity in people with mild-moderate Parkinson’s disease: a randomized controlled trial

**DOI:** 10.1186/s13063-023-07870-4

**Published:** 2024-01-22

**Authors:** Sandra G. Brauer, Robyn M. Lamont, John D. O’Sullivan

**Affiliations:** 1 School of Health and Rehabilitation Sciences, The University of Queensland, Qld, St Lucia, Australia; 2 UQ Centre for Clinical Research, Faculty of Medicine, The University of Queensland, Herston, Queensland Australia; 3 Department of Neurology, Royal Brisbane & Women’s Hospital, Herston, Queensland Australia

**Keywords:** Parkinson disease, Physical activity, Exercise, Physical therapy, Walking, RCT, Self-management

## Abstract

**Background:**

Physical activity levels are low in people with Parkinson’s disease (PD) and have proved difficult to increase with exercise programs alone. Intervention approaches that address both the capacity to engage in physical activity and self-management strategies to change and maintain exercise behaviours are needed to address this intractable issue.

**Methods:**

This will be an assessor-blinded, randomized controlled trial performed in Brisbane, Australia. Ninety-two people with mild-moderate PD will be randomly allocated to two groups: usual care, and a physiotherapy-led group exercise program combined with self-management strategies. In the intervention group, twelve, 80-min sessions will be conducted over 4 weeks in groups of up to 4 participants. The intervention will consist of circuit training including treadmill walking to target aerobic fitness, and activities targeting strength, balance, and gait performance. In addition, each session will also incorporate strategies focusing on self-management and behaviour change, augmented by the provision of a fitness activity tracker. Outcome measures will be collected at baseline (T1), immediately post intervention (T2) and at 6 months follow-up (T3). The primary outcome measure is free-living physical activity (average daily step count over 7 days) at pre (T1) and post (T2) intervention measured using an activPAL™ device. Secondary outcome measures captured at all time points include time spent walking, sedentary and in moderate intensity exercise over 7 days; spatiotemporal gait performance (step length, gait speed, endurance); health-related quality of life; and outcome expectations and self-efficacy for exercise.

**Discussion:**

Sustainability of gains in physical activity following exercise interventions is a challenge for most populations. Our incorporation of a chronic disease self-management approach into the exercise program including fitness tracking extends previous trials and has potential to significantly improve free-living physical activity in people with PD.

**Trial registration:**

This study has been prospectively registered in Australian and New Zealand Clinical Trial Registry (ACTRN12617001057370), registered on 19/07/2017. Available from www.anzctr.org.au/ACTRN12617001057370.aspx.

## Administrative information

**Table:** 

Title {1}	A physiotherapy exercise program with self-management approach to improve physical activity in people with mild-moderate Parkinson’s disease: a protocol for a randomized controlled trial.
Trial registration {2a and 2b}.	ACTRN12617001057370
Protocol version {3}	Version 1, Approved on December 9, 2016
Funding {4}	This work is supported by Wesley Medical Research, Principal investigators Brauer, O’Sullivan and Lamont.
Author details {5a}	1. School of Health and Rehabilitation Sciences, The University of Queensland, Qld, Australia 2. UQ Centre for Clinical Research, Faculty of Medicine, The University of Queensland, Herston, Queensland, Australia 3. Department of Neurology, Royal Brisbane & Women’s Hospital, Herston Queensland, Australia
Name and contact information for the trial sponsor {5b}	Lisa Kennedy, Director, Research Commercial Management The University of Queensland, St Lucia, Qld, 4072, Australia. Email: director.partnerships@research.uq.edu.au
Role of sponsor {5c}	The sponsor played no part in study design; collection, management, analysis, and interpretation of data; writing of the report; and the decision to submit the report for publication. The content is solely the responsibility of the authors and does not necessarily represent the official views of the University of Queensland.

## Introduction

### Background and rationale {6a}

Despite pharmacological intervention, people with Parkinson’s disease (PD) show a steady decline in physical functioning due to cumulative deficits in motor and non-motor impairments. Physical activity is important to maintain in people with PD, as it not only contributes to maintaining functions such as gait, balance in standing, and muscle strength, but these contribute to safe performance of activities of daily living and maintenance of independence. One area of decline is in physical activity. Levels of physical activity are lower in people with PD than healthy controls [1–3] and have been shown to decline overtime without intervention [4].

While a Cochrane review has found that exercise-based interventions can lead to short-term improvements in gait and function in people with PD [5], longer-term changes are difficult to achieve [6]. Physical activity behaviour is complex and multifactorial. Identified barriers to physical activity in people with PD include exercise self-efficacy and fear of falling [7]. Qualitative findings indicate that feedback and motivation influence decision making with regards to exercise participation in people with PD [8]. The largest study of intervention to improve physical activity in PD used personalized motivational coaching alone which was no more effective at increasing self-reported physical activity in sedentary people with PD than advice promoting safe movement [9]. Post hoc analysis however revealed that those participants with greater levels of physical fitness and better walking ability demonstrated greater increases in physical activity than the control group [10]. Intervention to improve levels of physical fitness and walking ability may therefore be an important component of promoting physical activity behaviour change in this population.

Randomized controlled trials have demonstrated that regular aerobic exercise is achievable for people with PD and can contribute to improved fitness [11, 12] and slowing the progression of symptoms over 3 to 6 months [13, 14]. Prescribed exercise programs including stationary cycling, stationary trainers and treadmill walking have all shown positive results when performed for at least 30 min, 3 times each week at moderate and high intensities, with high-intensity training proving more beneficial for slowing progression of primary impairments [13]. The ongoing engagement in physical activity following a prescribed exercise program is a challenge however, and education about and performance of exercise alone may not be sufficient to change behaviour. Personal factors such as self-efficacy for exercise, motivation, or fear of falling, and environmental factors such as social support, accessibility or poor weather are often barriers identified to start or remain engaged in exercise in people with PD and should be addressed [7, 15].

Several self-management approaches for people with chronic disease can together address these factors. The Health Action Process Approach [16] consists of two phases—a motivational phase where beliefs drive activities, and a volitional phase where self-regulatory skills and strategies (action planning, coping planning, recovery self-efficacy and social support) are employed to translate intentions into action [17]. Goal Setting Theory [18] underpins goal development which drives the volitional phase of action. Finally, self-determination theory can also be applied to intervention design to enhance understanding, motivation and thus engagement with an intervention [19], addressing the need for competence, autonomy and relatedness. Few studies have evaluated the impact of self-management approaches to improve physical activity or exercise update in people with PD [20–22]. These have varied in design, population studied, aim and type of intervention, and outcomes measured, with heterogenous results found to date.

### Objectives {7}

This trial aims to determine if a small group physiotherapist-led exercise program combined with a self-management approach is more effective than usual care at promoting increased physical activity in people with mild to moderate Parkinson’s disease. Data will be collected at baseline (T1), immediately following intervention (T2, +4 weeks) and 6 months after intervention (T3, +26 weeks).

Small group physiotherapist-led exercise sessions will be used to address participants’ physical fitness, confidence and motivation to get going and keep going with exercise. Physical fitness will be addressed by ensuring that a primary component of the group exercise will be using treadmill training to ensure participants work at a moderate-high heart rate (60–80% HRR) for 30 min every session. This trial extends previous work by using this training to explicitly improve exercise self-efficacy (or belief they can exercise) and by adding it to a self-management framework to help people with PD keep exercising.

This project aims to determine if a group program of exercise aimed to increase physical fitness and gait performance incorporated with a self-management approach results in a greater and more sustained improvement in free-living physical activity (steps/day) and secondary outcomes than usual care.

### Trial design {8}

This study is a two-arm, parallel group, assessor-blinded randomized controlled superiority trial. Randomization will be performed in variable block permutations by an offsite person independent to the study with a 1:1 allocation ratio.

## Methods: participants, interventions and outcomes

### Study setting {9}

The study is planned to be conducted in multiple sites within the metropolitan south-east region of Queensland, Australia, including a university gait laboratory, and in the gym of hospital or community-based physiotherapy outpatient services. This region has a population size of approximately 3.8 million and includes the capital city, Brisbane.

### Eligibility criteria {10}

The inclusion criteria for participants are as follows: a diagnosis of idiopathic PD confirmed by a neurologist; Modified Hoehn & Yahr stage I to III; living in the community; able to attend assessment and intervention sessions; a Montreal Cognitive Assessment Scale (MoCA) score of >23/30 [23]; and willing and able to provide consent. Exclusion criteria include people who have had surgical management of their PD (e.g. deep brain stimulation); a diagnosis of any other neurological disorders, uncorrected sensory deficits, or medical conditions that may limit their ability to safely exercise; and people who report that they are already meeting physical activity guidelines for older adults (exercising for >150 min/week at a moderate intensity).

### Recruitment {15}

Calls for participants will be made via local neurologists, physiotherapists, Parkinson’s support organizations and social media. All study procedures will be discussed individually with volunteers by a member of the research team, who will clarify any questions, before screening for eligibility.

### Who will take informed consent? {26a}

Following this, informed written consent will be signed voluntarily by participants and witnessed by the researcher responsible for gaining informed consent.

### Additional consent provision for collection and use of participant data and biological specimens {26b}

No biological specimens will be collected as part of this trial. At the completion of the trial, participants will be provided with an opportunity to provide consent to be contacted by trial investigators for any future studies.

## Interventions

### Explanation for the choice of comparators {6b}

Usual care for people with mild-moderate PD in Australia includes advice and education about exercise and opportunities to exercise which can come from multiple disciplines including medical professionals (e.g. neurologists), physiotherapists and exercise professionals. Including a comparator of usual care allows us to evaluate the real-world impact of implementing the trial intervention in the current health care setting.

### Intervention description {11a}

Participants in the intervention group will take part in twelve, small group (max 4 participants) structured exercise sessions over 4 weeks, each 75–80 min in duration, led by a registered physiotherapist trained to provide the intervention. Exercise sessions will include a short group warm up followed by a structured circuit of 30 min of aerobic exercise training, lower limb progressive resistance strength training, balance and gait retraining, followed by a cool down session. Aerobic exercise training will occur primarily via walking on a treadmill at an intensity of 60–80% of heart rate reserve determined using the Karvonen formula (i.e. Target Heart Rate = ((max HR − resting HR) × 60–80%) + resting HR). As exercise tolerance improves, walking speed and incline of the treadmill will be increased to maintain this level of intensity over the 12 sessions [12].

Progressive resistance strength training will target the hip and knee flexors and extensors, hip abductors and ankle plantarflexors. Three sets of eight repetitions of each exercise will be performed at 40–60% of the participants one repeat maximum (1RM), with resistance increased by 5% when participants are able to achieve 10 repetitions at 60% of their 1RM [24]. Resistance exercises will be performed using both variable resistance equipment and functional resistance exercises [25] depending on what is available to the participant after the 4 weeks of supervised exercise to maximize carryover to the home environment. Individualized balance and gait retraining exercises will be prescribed as indicated for each participant incorporating the principles of movement strategy training [26] and highly challenging balance retraining [27].

The self-management approach will be delivered during the same 4-week period that participants are participating in the exercise groups. It will involve 5–10-min sessions, delivered individually to the participants by the physiotherapists prior to or during the exercise sessions. Behaviour change techniques used in this self-management approach will include education, behavioural instruction, self-monitoring, goal setting and goal review, feedback, problem solving, action planning and coping planning to encourage initiation and maintenance of physical activity. At the first exercise session, participants in the intervention group will receive information and a workbook to encourage self-monitoring of physical activity and guide participants through the process of setting short- and long-term goals as well as formulating action plans and coping strategies. During the 4 weeks of exercise sessions and the following 6 months, participants will also be provided with a Garmin Vivosmart fitness tracker that monitors and sets goals for physical activity (number of steps / day), and records heart rate, and distance travelled. Participants will be instructed in how to use the device and associated application/software, and their self-monitoring ability will be tracked during the 4 weeks with the physiotherapist. During the follow-up period participant activity will be monitored monthly via the online Garmin platform. If a reduction in activity level is noted the participant will be contacted by phone to check in and to discuss their physical activity goals and achievements.

### Criteria for discontinuing or modifying allocated interventions {11b}

All participants will receive their allocated intervention unless the participant chooses to discontinue. In the event of illness or injury related or unrelated to the trial, the specific exercise intervention may need to be modified. All data will be analyzed on an intention to treat basis, where participants will be analyzed according to their original group assignment.

### Strategies to improve adherence to interventions {11c}

Strategies to improve adherence to the intervention are inherent in the self-management component of the intervention. There are no additional strategies.

### Relevant concomitant care permitted or prohibited during the trial {11d}

All participants will continue with their usual pharmaceutical care during the trial. There is no restriction on accessing external therapy or exercise groups.

### Provisions for post-trial care {30}

In the event of an adverse event or harm because of their participation in the trial will be provided with relevant ongoing care under the trial insurance.

### Outcomes {12}

The primary outcome measure is free-living physical activity (average number of steps per day) at post intervention (T2) assessment collected via an activity monitor (activPAL™) that will be worn for seven complete 24-h periods.

Secondary outcomes will include (a) free-living physical activity (average number of steps per day) at follow-up (T3); (b) additional measures of free-living physical activity (average daily time spent walking, average daily time spent sedentary) at each assessment time point; (c) gait endurance measured via the 6-min walk test at each assessment time point; (d) spatiotemporal gait parameters (step length, gait speed) during single and dual task conditions over 8 m using a GAITrite™ electronic walkway at each assessment time point; and (e) health-related quality of life (QOL) at each assessment time point; will be measured using the 39-item Parkinson’s disease questionnaire (PDQ-39).

### Participant timeline {13}

**Fig. 1:**
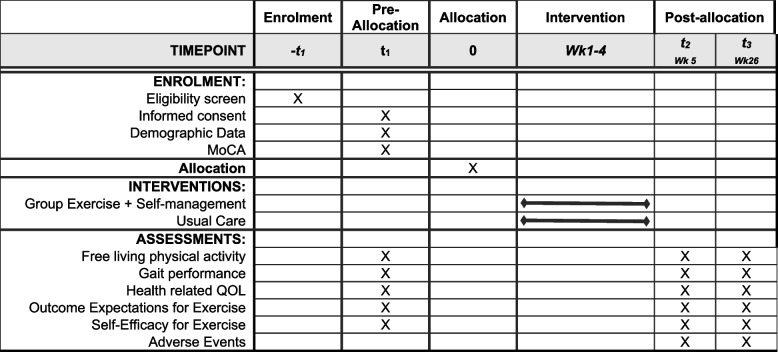
Schedule of enrolment, interventions, and assessments

**Fig. 2:**
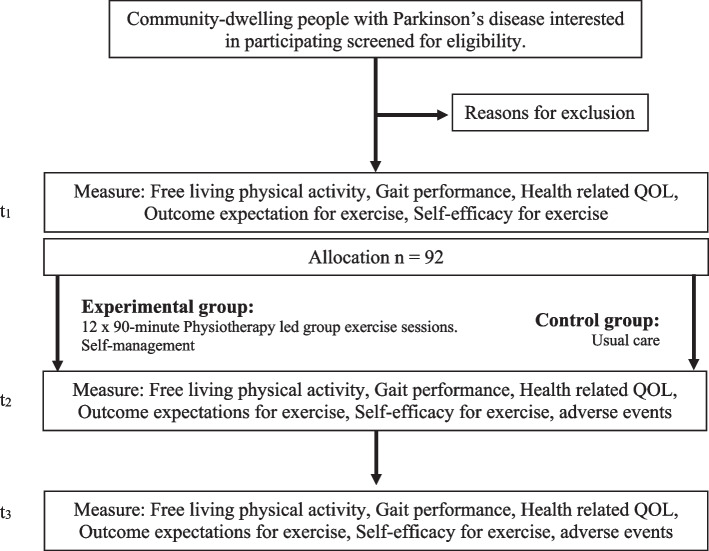
Participant flow

The participant flow is shown in Figs. 1 and 2. All participants will be engaged in the study for 6 months. Assessments will be conducted at baseline (T1 = week 0), post intervention (T2 = +week 5) and at 6 months follow-up (T3 = week 26). Allocation to intervention will occur on completion of the baseline assessment.

### Sample size {14}

The required sample size has been determined to be 92 participants (46/group) to detect a 22% increase in the number of steps/day immediately following the 4-week intervention. The proportion of change is based on previous studies of people with mild-moderate idiopathic PD who volunteered for exercise trials [1]. This determined a need for 41 participants per group. A conservative 12% allowance for withdrawals was included based on previous exercise trials in people with Parkinson’s disease [9]. Sample size was calculated using SPSS.

## Assignment of interventions: allocation

### Sequence generation {16a}

Computerized random numbers were generated in variable block permutations of 4 or 6 using Matlab™.

### Concealment mechanism {16b}

To conceal randomization, an independent offsite researcher will create the schedule and prepare consecutively numbered opaque envelopes with the group allocation of each participant sealed inside and locked in a location inaccessible to the assessors.

### Implementation {16c}

After completing all baseline assessment requirements, participants will be contacted by phone to receive their group allocation. Participants allocated to the intervention group are scheduled to attend the group exercise sessions. The allocation phone call is made to participants by a member of the research team involved in intervention, but not responsible for any assessment.

## Assignment of interventions: blinding

### Who will be blinded {17a}

The study is a single-blind design where assessors are blinded to participant allocation. The randomization schedule has been prepared and concealed by a researcher separate to this research study. Allocation is revealed following baseline assessment and is revealed to participants by a researcher who is independent to the assessment of outcomes (one of the intervention physiotherapist/researchers). The randomization envelopes will be used to reveal each participant allocation in turn, to ensure that the researchers who provide the intervention remain blinded to all future allocations. Participants are reminded at the time of allocation and before their post and follow-up assessments that the assessor cannot be told who has been allocated to which group. Participants in the intervention group are additionally asked not to wear the activity monitoring watch on the day of their assessments. Data analysis will be completed by an individual blinded to group allocation.

### Procedure for unblinding if needed {17b}

There is no forseeable reason for the assessor to be unblinded to participant allocation. Members of the research team independent of the assessment of outcomes will be responsible for managing any event that may require knowledge of the participant allocation.

## Data collection and management

### Plans for assessment and collection of outcomes {18a}

All assessments will be conducted by a registered physiotherapist who has had specific training in the conduct of all outcome measures.

## Physical activity

Participants will wear an activity monitor (activPAL™) fixed to the anterior thigh using a waterproof adhesive dressing continuously for nine consecutive days at each assessment point. The activPAL™ is a lightweight electronic logger that contains a microprocessor, sensing element (accelerometer, gyroscope), recording element, associated electronics and power supply. It has demonstrated excellent accuracy measuring step count during treadmill and outdoor walking, with some terrain challenges and attention demands, at a variety walking speeds in people with PD [28]. The activPAL™ has been used to quantify sedentary behaviour [29] and ambulatory activity [3, 30] in people with PD.

Step count data from the seven complete 24-h (midnight to midnight) periods will be averaged to give steps/day. Additional measures of free-living physical activity (e.g. average daily time spent walking, average daily time spent sedentary, total minutes/week spent in moderate intensity exercise, time spent in bouts of activity) will be drawn from the 7 days of activPAL™ recording at each of the three assessment time points. The proportion of people meeting American College of Sports Medicine physical activity guidelines with respect to volume of physical activity will also be calculated (Garmin vivoSMART).

## Gait and balance

Gait endurance will be measured using the 6-min walk test which has excellent test-retest reliability (ICC = 0.96) and established minimal detectable change values in this population [31]. Functional mobility and balance will be measured using the Timed up and go (TUG) test with and without added dual tasks [32]. Gait speed and step length will be measured with and without added dual tasks using the 8m instrumented GAITrite™ walkway and existing testing protocols [33].

## Quality of life

Health-related quality of life will be measured using the 39-item Parkinson’s disease questionnaire—PDQ-39 [34]. It will be collected at all three time points with an interviewer present to clarify questions or assist with writing if required.

## Participant characteristics

Participant characteristics collected at baseline will include demographic data (age, sex, years since diagnosis), medical history and general health status. To characterize their Parkinson’s disease, at every time point severity of PD will be rated using the Hoehn & Yahr Stage [35]; levodopa equivalent dose will be calculated from their prescribed medication; and the Movement Disorders Society MDS-Unified Parkinson’s disease rating scale motor subsection III [36] and the Freezing of Gait questionnaire [37] will be completed. Exercise self-efficacy will be measured at post and follow-up assessments using the Outcome Expectations for Exercise (OEE) Scale [38] and the Self-efficacy for Exercise Scale (SEEs) [39].

The occurrence and details of any adverse events (e.g. falls), experienced since the last assessment session, will be recorded at the post intervention and follow-up assessments.

### Plans to promote participant retention and complete follow-up {18b}

All data will be managed with an intention to treat philosophy, including any who deviate from the allocated intervention. Once participants are enrolled in the study, the research team will make every reasonable effort to follow them for the entire study period, including reminding them of upcoming data collection appointments. Participant retention through to follow-up will be optimized by regular contact throughout the intervention and follow-up period for those in the intervention group. Participants in the control group will be contacted between time points to schedule appointments and provide reminders. Any deviations from the study protocol and the trial’s time plan (including withdrawal) or from the intervention will be documented.

### Data management {19}

Following each assessment, data will be entered into a computer spreadsheet saved on a protected computer server within the university. Accuracy of data entry will be audited twice yearly by people external to the research team. Paper-based data files will be secured in a lockable cabinet in the locked office of the research team. All data will be retained for 15 years after completion of the research, to comply with Australian Government legislation.

### Confidentiality {27}

All written data will be maintained in a locked filing cabinet in the locked office of one of the chief investigators. Electronic data will be stored on a password-protected computer.

### Plans for collection, laboratory evaluation and storage of biological specimens for genetic or molecular analysis in this trial/future use {33}

As seen in the “Additional consent provision for collection and use of participant data and biological specimens {26b}” section above, no biological specimens will be collected as part of this trial.

## Statistical methods

### Statistical methods for primary and secondary outcomes {20A}

To determine if there are differences between groups in physical activity outcomes post intervention, linear mixed models will be performed investigating group (intervention vs. control), time (baseline, post, follow-up) and group × time interactions for all variables. Baseline participant characteristics will be compared between groups and those with any differences included as covariates. Post hoc univariate testing will be undertaken if main effects and interactions are significant.

### Interim analyses {21b}

No interim analysis will be performed.

### Methods for additional analyses (e.g. subgroup analyses) {20b}

No additional or subgroup analyses will be performed as part of the trial

### Methods in analysis to handle protocol nonadherence and any statistical methods to handle missing data {20c}

The analysis to handle protocol nonadherence will follow intention to treat principles. Linear mixed models permit inclusion of all cases even if data could be missing from some timepoints. Missing data will be ignored, rather than imputed.

### Plans to give the full protocol, participant-level data and statistical code {31c}

The full protocol is registered with the ANZCTR and publicly available via their platform. The data sets used and analyzed for this study will be available from the corresponding author on reasonable request.

## Oversight and monitoring

### Composition of the coordinating center and trial steering committee {5d}

The Centre for Neurorehabilitation, Ageing and Balance Research within the School of Health and Rehabilitation Sciences at the University of Queensland is the coordinating center. Prof Sandra Brauer is the center director who will be responsible for design and conduct of the trial, organizing committee meetings, managing reporting requirements and publication of study results. All investigators and the trial manager are members of the trial management committee who are responsible for reviewing study progress, data and administration management and reviewing trial conduct. This team has all completed the Good Clinical Practice certification in Australia. They meet fortnightly with trial progress reviewed and reported to the funding body and ethics annually.

### Composition of the data monitoring committee, its role and reporting structure {21a}

For this protocol, a DMC is not needed as this is a low-risk intervention (exercise at this level is recommended for all people with PD) and it is not required by the ethics committee and regulatory authorities.

### Adverse event reporting and harms {22}

The risk of harm during assessment, or intervention is small, mitigated by the presence of a physiotherapist experienced in working with people in this population, in a clinical exercise environment and experienced in exercise prescription. Any adverse events that occur during or between intervention sessions will be recorded by the physiotherapist supervising the intervention. At each scheduled assessment time point participants will be asked if any adverse events (related or unrelated to the study) have occurred since the previous assessment. All participants will be asked to contact the researcher responsible for intervention, if they experience any falls, injury or illness that impacts on their movement or exercise during the follow-up period. Minor adverse events and their outcomes are recorded as part of routine data collection. Major adverse events will be reported to the principal investigator and on to the relevant ethics committee as per the reporting requirements of the ethics committee. All harms that occur during the participant’s enrolment in the study will be graded using the Common Terminology Criteria for Adverse Events (CTCAE) Version 5.0 (November 27, 2017) and reported in the results manuscript.

### Frequency and plans for auditing trial conduct {23}

Trial conduct will be audited by reviewing data quality every 6 months. This will include review of recruitment rates, source documents, data collection, data entry and adverse events. Reports including recruitment rate, impacts to the trial (e.g. impact of COVID-19), deviations from the protocol and adverse events will be prepared for annual reports to the submission to the funding body and ethics committee. Any project approved by the University Human Research Ethics Committee may be audited at random.

### Plans for communicating important protocol amendments to relevant parties (e.g. trial participants, ethical committees) {25}

Any protocol amendments will be agreed by the trial management committee and must be submitted and approved by the relevant ethics committees. They will also be reported to the funding agency and the clinical trial registry updated.

### Dissemination plans {31a}

The trial results will be disseminated to health care professionals and the scientific community via both publication of the results in relevant journals and presentation of the results at conferences. Results will be included in the completion report for the ANZCTR and the funding body. A summary of the results will be disseminated directly to all participants on completion of data collection.

## Discussion

This trial evaluates a multi-modal intervention designed to improve the volume of free-living physical activity in people with Parkinson’s disease. It uses a novel approach of combining supervised group exercise intervention with behavioural counselling and the provision of an activity tracker to facilitate self-management.

It is expected that this combination of structured exercise and self-management will be effective in firstly improving average daily step count immediately post training as an indication of free-living physical activity but will also importantly give people with PD the training and skills to maintain these improvements for the following 6 months. The intervention has been designed to enhance participants’ motivation to engage in physical activity in several ways. Participants will gain evidence of their competence to exercise via feedback from their physiotherapist and other participants in their exercise sessions, in addition to feedback from their fitness tracker. The self-management approach builds self-regulatory skills and strategies to enable people with PD to create goals related to physical activity and make informed decisions about their activity outside the training sessions, thus building autonomy. In addition, the ongoing use of accelerometers allows participants to track their own activity levels, rather than relying on research staff. Providing the intervention in group sessions facilitates social engagement and support, and helps people feel part of a group, which can contribute to motivation.

Results of this study are likely to add to the growing body of knowledge on the development of approaches to optimize walking in people with PD. Identifying interventions that can assist people with PD to increase their levels of physical activity has the potential to maintain health, walking ability and quality of life for longer.

## Limitations

The authors acknowledge the following limitations: lack of a cost-effectiveness analysis, and limited patient and public involvement in the design of the intervention.

## Trial status

The trial is ongoing and participant recruitment has not been completed. Protocol version 1.0 was approved on Dec 9, 2016. Recruitment began on August 25, 2017. Significant delays to recruitment have occurred from February 2020 due to COVID-19. Approximate date when recruitment will be completed: September 2023. Significant time pressure on the investigative team during COVID-19 also resulted in delays to the completion of this manuscript.

## Abbreviations

PD: Parkinson’s disease
HR: Heart rate
HRR: Heart rate reserve
MoCA: Montreal Cognitive Assessment
1RM: One Repeat Maximum
QOL: Quality of life
PDQ-39: 39-Item - Parkinson’s disease questionnaire
OEE: Outcome Expectations for Exercise Scale
SEEs: Self-efficacy for Exercise Scale
